# Icaritin inhibits breast cancer through activating MT1X to promote ferroptosis and synergizing with NF-κB pathway suppression

**DOI:** 10.3389/fonc.2026.1868122

**Published:** 2026-07-10

**Authors:** Fenglin Zhan, Dachang Qiu, Chenyu Wang, Ning Sun

**Affiliations:** 1 Wuxi School of Medicine, Jiangnan University, Wuxi, China; 2 Department of Nuclear Medicine, The First Affiliated Hospital of University of Science and Technology of China (USTC), Division of Life Sciences and Medicine, University of Science and Technology of China, Hefei, Anhui, China; 3 School of Medical Imaging, Wannan Medical College, Wuhu, China

**Keywords:** breast cancer, Epimedium-derived compounds, ferroptosis, icaritin, MT1X, NF-κB signaling

## Abstract

**Introduction:**

Icaritin (ICT) is a natural isoprenylated flavonoid compound extracted from the traditional Chinese herbal medicine *Epimedii Folium*. Metallothioneins (MTs) are a group of low-molecular-weight, cysteine-rich proteins that play crucial roles in oxidative stress, metal homeostasis, and cancer drug resistance. The anticancer effect and detailed regulatory mechanism of ICT in breast cancer remain to be fully clarified. This study aims to investigate the anti-tumor activity of ICT in breast cancer and explore its underlying molecular mechanism.

**Methods:**

*In vitro* assays were performed in breast cancer cell lines to evaluate cell viability, intracellular Fe^2+^ levels, reactive oxygen species (ROS) production, and protein expression of the ferroptosis-associated antioxidant molecules SLC7A11 and GPX4 following ICT treatment. A ferroptosis inhibitor was employed to confirm the causal role of ferroptosis in ICT’s efficacy. Key target genes regulated by ICT were screened via transcriptome sequencing, and functional validation was conducted through MT1X overexpression to assess its impacts on NF-κB pathway activation, cell cycle distribution, and the SLC7A11/GPX4 axis. A breast cancer xenograft mouse model was established to verify the in vivo anti-tumor effect and mechanism of ICT.

**Results:**

ICT induced ferroptosis and suppressed viability in breast cancer cells, as evidenced by intracellular Fe^2+^ accumulation, elevated ROS levels, and downregulated SLC7A11 and GPX4. This anti-tumor effect was reversed by a ferroptosis inhibitor, confirming ferroptosis as the key mechanism of ICT. Transcriptomic analysis and functional validation showed that ICT markedly upregulated MT1X. MT1X overexpression inhibited NF-κB signaling via reducing IκBα and p65 phosphorylation, induced G1 cell cycle arrest, and suppressed the SLC7A11/GPX4 antioxidant axis, synergistically enhancing ICT’s anti-proliferative activity. *In vivo*, ICT-mediated MT1X upregulation promoted ferroptosis and inhibited tumor growth.

**Discussion:**

This study elucidates that ICT exerts anti-breast cancer effects by activating MT1X to induce ferroptosis and cooperatively inhibit the NF-κB signaling pathway. Our findings reveal a novel molecular mechanism of ICT against breast cancer, and provide a new therapeutic target and experimental basis for breast cancer treatment.

## 1 Introduction

Breast cancer remains the most frequently diagnosed malignancy in women and continues to be a leading cause of cancer-related death worldwide (1, 2). Recent epidemiological data published in *Nature Medicine* reported approximately 2.3 million new cases and 670,000 deaths globally in 2022. Alarmingly, projections suggest that by 2050, the annual incidence could escalate to 3.2 million, with mortality approaching 1.1 million cases (3). These figures underscore a pressing need to revisit current prevention and treatment strategies. Although considerable progress has been made in molecular diagnostics and targeted therapies, many patients still face challenges due to late-stage diagnosis, drug resistance, and metastasis (4). As such, there is an urgent need for innovative, mechanism-based treatment approaches that can effectively complement existing clinical strategies.

Ferroptosis is a distinct form of iron-dependent programmed cell death, marked by intracellular iron accumulation and lipid peroxidation driven by excessive ROS production (5–7). Notably, cancer cells often exhibit dysregulated iron metabolism and elevated iron dependency for proliferation, a phenomenon termed “iron addiction” (8). This makes them particularly susceptible to ferroptotic death under iron-rich conditions (9). A growing body of evidence suggests that inducing ferroptosis can overcome resistance to conventional therapies in cancers such as breast, ovarian, and hematological malignancies (10, 11). In breast cancer, ferroptosis activation not only impairs tumor cell viability but also enhances chemosensitivity and suppresses metastasis by modulating epithelial-mesenchymal transition (EMT) and the tumor microenvironment (12).

**Figure 1:**
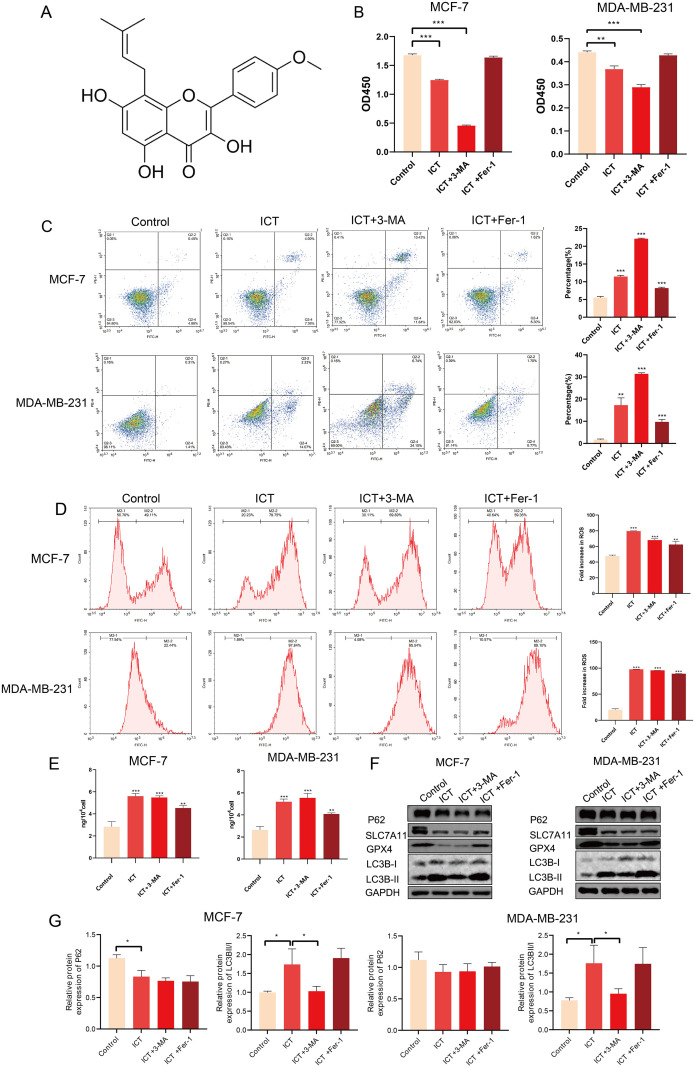
ICT induces ferroptosis in breast cancer cells. **(A)** The molecular structure formula of ICT. MCF-7 and MDA-MB-231 cells were treated with blank control medium (Control), 20μM ICT, 20μM ICT + 10mM 3-MA, 20μM ICT + 1μM Fer-1 for 48 hours. **(B)** Cell viability changes in different treatment groups were detected by CCK-8 assay. **(C)** apoptosis levels in each group were detected by flow cytometry. **(D)** Detection of ROS levels by DCFH-DA fluorescence probe method. **(E)** Detection of Fe2^+^ content by iron ion colorimetric method. **(F, G)** Protein expression of P62, SLC7A11, GPX4, LC3B-I and LC3B-II in BC cells after ICT treatment. GAPDH was used as a control substance, and the representative column diagrams showed results of relative protein expression. Data was represented as the mean ± SD of three independent experiments. *p<0.05; **p<0.01, ***p<0.001.

In recent years, growing interest has focused on the anticancer properties of natural compounds. Several bioactive agents, including resveratrol and curcumin, have been shown to suppress breast cancer cell proliferation by activating autophagy and ferroptosis pathways (13), providing important mechanistic insights for natural product-based antitumor research. Icaritin is the monomeric active constituent derived from the traditional Chinese medicinal herb Epimedii Folium. Its structure features a fused benzopyran ring skeleton and it is classified as an isoprenylated flavonoid derivative with a broad spectrum of biological activities (**Figure 1A**), has been shown to possess a wide spectrum of biological activities, including antioxidant, anti-inflammatory, immunoregulatory, and neuroprotective effects (14, 15). In the context of oncology, ICT has emerged as a promising agent due to its ability to modulate various oncogenic signaling pathways involved in cell proliferation, apoptosis, angiogenesis, and metastasis (16–18). Tang et al. found *in vitro* experiments highlight ICT’s role in the crosstalk between autophagy and apoptosis, enhancing apoptosis through autophagy inhibition (19). Additionally, ICT can also induce ferroptosis in colorectal cancer cells by activating mitochondrial dysfunction (20). However, there is no report on whether ICT can induce ferroptosis in breast cancer cells. In this study, we revealed a new mechanism by which ICT inhibits the growth of breast cancer, that is, ICT down-regulates the expression of SLC7A11 and GPX4 through the MT1X, promotes the accumulation of Fe^2+^ and the production of ROS, thereby inducing ferroptosis in breast cancer cells and inhibiting tumor progression. Based on these results, it may be concluded that therapeutic interventions mediated through the use of ICT-induced ferroptosis can potentially provide a promising strategy for the treatment of breast cancer.

## 2 Materials and methods

### 2.1 Cell culture and treatment

MCF-7 and MDA-MB-231 breast cancer cells, along with MCF-10A normal breast epithelial cells, were obtained from the Shanghai Cell Bank (Shanghai, China). These two breast cancer cell lines represent the two most common clinical subtypes with distinct biological behaviors and therapeutic responses: MDA-MB-231 is a highly invasive triple negative breast cancer (TNBC) cell line, while MCF-7 is a poorly aggressive estrogen receptor positive (ER+) breast cancer cell line (21). After revival, MCF-7 and MDA-MB-231 cells were cultured in 1640 medium (C11875500BT, Gibco, USA), while MCF-10A cells were grown in DMEM medium (C11965500BT, Gibco). All culture media were enriched with 10% fetal bovine serum (A3160802, Gibco) and 1% penicillin-streptomycin solution (15140122, Gibco). In the ICT (SML 0551, Sigma, USA) treatment group, cells were subjected to 20 μmol/L ICT and incubated for 48 hours before being used in further experiments.

### 2.2 Cell transfection

Cells in the logarithmic growth phase were transfected when the cell fusion rate was 40% - 60%. For transient transfection, cells were transferred to serum-free medium and then transfected with the MT1X overexpression plasmid (pCDH-CMV-MCS-EF1-CopGFP-T2A-Puro-MT1X) or the corresponding empty vector control plasmid (pCDH-CMV-MCS-EF1-CopGFP-T2A-Puro) using Lipofectamine 2000 (11668500, Thermo Fisher Technologies, USA). Transfection efficiency was verified by quantitative real-time PCR (qPCR) and Western blot analysis. All subsequent experiments were performed 24 hours after transfection.

### 2.3 Cell proliferation detection

Cells in the exponential growth phase were selected and prepared according to the specific needs of the experiment. The cell suspension from each group was adjusted to a density of 2×10^4 cells/ml, and 100 μl samples were transferred into a 96-well plate. These plates were then cultured at 37 °C under 5% CO_2_ for 24 hours to enable pre-culturing. For dose response experiments, cells were treated with ICT at a series of concentrations (0, 5, 10, 20, 40 μmol/L) for 48 hours. For subsequent functional experiments, cells were treated with 20 μmol/L ICT or an equal volume of vehicle control for 48 hours. Afterward, 10 μl of CCK8 solution (B1099, Bioogenetech, China), was gently added to each well, ensuring no bubbles were introduced, as they could interfere with optical density readings. The plates were incubated again for an additional two hours. Finally, the absorbance was measured at 450 nm using a microplate reader (Infinite F50, Tecan, Switzerland). IC50 values were calculated using GraphPad Prism 8.0 software. All experiments were performed in triplicate and repeated three times independently.

### 2.4 Cell apoptosis detection

When the cells in each experimental group of 6-well plates reached a coverage of approximately 70%, the supernatant was collected, adherent cells were digested with trypsin (B2048, Bioogenetech) and the cells were resuspended in complete medium. The supernatant cells and adherent cells were collected in the same 5 mL centrifuge tube, with three replicate wells for each group (to ensure sufficient cells for analysis, the cell number was ≥ 5×10^5^ per treatment). The cells were centrifuged at 1000g for 5 minutes, the supernatant was discarded, and the cells were gently resuspended in PBS (C10010500BT, Gibco). We employed a commercial apoptosis detection kit (C1062S, Beyotime, China) following the manufacturer’s protocol. Centrifuge 1000g for 5 minutes, discard the supernatant, and add 195 μl of Annexin V-FITC binding buffer to gently resuspend the cells. Add 5 μl of Annexin V-FITC and gently mix. Add 10 μl of propidium iodide staining solution and gently mix. Use aluminum foil to shield from light and incubate at room temperature (20-25 °C) for 10–20 minutes, resuspending the cells 2–3 times during the incubation process. Proceed to detection on the flow cytometer (2060R, NovoCyte, USA).

### 2.5 Cell cycle detection

Cells from each experimental group were harvested at approximately 70% confluence. The cell cycle was analyzed using a commercial cell cycle detection kit (B1011, Bioogenetech). Briefly, cells were digested with trypsin, collected by centrifugation at 1000 g for 3 minutes, and the supernatant was carefully removed. The cell pellet was washed with 1 mL of ice-cold PBS, then fixed in 1 mL of ice-cold 70% ethanol under gentle mixing and stored overnight at 4 °C. After fixation, cells were resuspended in 0.5 mL of propidium iodide staining solution and incubated in the dark at 37 °C for 30 minutes. Samples were analyzed on a flow cytometer using an excitation wavelength of 488 nm to detect red fluorescence and light scattering. Data were processed with dedicated software to assess DNA content and light scattering characteristics.

### 2.6 Western blotting

Extracted total protein using RIPA lysis buffer (containing PMSF, R0010, Solarbio, China), and then quantified the total protein using a BCA quantification kit (P0009, Beyotime) Performed SDS-PAGE electrophoresis (PG112, Yamay Bio, China), transfer, and blocked the transfer membrane. Incubated with primary antibodies P62 (18420-1-AP, Proteintech, China), SLC7A11 (26864-1-AP, Proteintech), GPX4 (ab125066, Abcam, UK), LC3B (381544, ZENBIO, China), and GAPDH (60004-1-Ig, Proteintech). Incubated overnight at 4 °C, then washed three times with PBS. Incubated with secondary antibodies. After secondary antibody incubation, detected on a chemiluminescence imaging instrument (SH-Advance 523, shenhuabio, China).

### 2.7 Measurement of ROS levels

Intracellular reactive oxygen species (ROS) levels were detected using a commercial ROS assay kit (CA1410, Solarbio) according to the manufacturer’s instructions. Briefly, a 10 μmol/L DCFH-DA working solution was prepared by diluting the stock solution in serum-free medium at a ratio of 1:1000. After collection, cells were resuspended in the diluted DCFH-DA solution at a density of 1–2 × 10^6^ cells/mL and incubated at 37 °C for 20 minutes in a cell culture incubator. The suspension was gently mixed every 3–5 minutes to ensure uniform probe exposure. Following incubation, cells were washed three times with serum-free medium to remove any unincorporated DCFH-DA. Cells were then either stimulated with a ROS-inducing positive control or with the test drug; alternatively, they were divided into several equal groups for respective treatments. The positive control was confirmed to induce a significant increase in ROS within 20–30 minutes after stimulation.

### 2.8 Determination of Fe²^+^ content

Intracellular Fe²^+^ levels were determined using a commercial iron content assay kit (BC5315, Solarbio). Briefly, 5 × 10^6^ cells were resuspended in 0.5 mL of extraction solution and subjected to ultrasonic disruption on ice (200 W, 3 s pulse-on, 7 s pulse-off, 30 cycles). The homogenate was then centrifuged at 8000 g for 10 minutes at 4 °C. The resulting supernatant was collected for subsequent Fe²^+^ analysis.

### 2.9 RNA sequencing

The nucleic acid samples extracted from the NC group (which received no treatment) and the ICT group (treated with 20 μmol/L for 48 hours) were cut into small fragments. These fragmented nucleic acids underwent end repair, A-terminal modification, and adapter ligation processes to construct sequencing libraries. The nucleic acid fragments in the sequencing libraries were amplified by PCR and then sequenced and analyzed using Illumina NovaSeq 6000 (Illumina, CA, USA). Gene Ontology (GO) enrichment and KEGG pathway analyses of all differentially expressed genes were conducted with the ClusterProfiler package, following the approach established by Kanehisa et al (22). The resulting data were then identified and visualized.

### 2.10 PPI network

Based on the target gene MT1X, the top 50 proteins associated with the target protein were obtained from the STRING (version 12.0) database. A protein-protein interaction network was constructed using the obtained proteins (1 target protein + 50 associated proteins).

### 2.11 qRT-PCR

Initially, high-quality RNA is isolated from the sample to maintain both its integrity and purity. The extracted RNA was then converted into complementary DNA (cDNA) through reversed transcription using reverse transcriptase. Subsequently, targeted amplification of the cDNA was performed via PCR technology, utilizing appropriate primers and reaction conditions to acquire the desired fragment (sequences provided in **Supplementary Table 1**). The PCR process employed SYBR Premix Ex Taq™ (TaKaRa) and was carried out on the ABI StepOne™ Real-Time PCR System (Applied Biosystems, CA).

### 2.12 Establishment of tumor xenografts in nude mice

Female BALB/c nude mice (6–8 weeks old, 17–21 g) were obtained from the Shanghai Slike Laboratory Animal Co., Ltd. Taked MT1X-OE or NC cells that were in the logarithmic growth phase, digested them with trypsin, then centrifuged and resuspended them. Adjusted the cell density to 1×10^7^ cells/ml (with a matrix gel mixture ratio of 1:1), and then injected 200 microliters of the cell suspension (approximately 2×10^6^ cells per mouse) subcutaneously into the right axilla of nude mice(n = 5 per group). Observed the inoculation site every day, and measured the long diameter (L) and short diameter (W) of the tumor using a vernier caliper. Calculated the tumor volume: V = (L×W²)/2. Started drug treatment when the tumor volume reaches 100 mm³ (approximately 7–10 days) and continued for 21 days, administering the drug at fixed times each day. Mice in the treatment group received intraperitoneal injection of 10 mg/kg ICT, a dose shown to be effective and non-toxic in female BALB/c mouse models in previous studies (23). Control group: the same volume of normal saline. 24 hours after the last administration, all mice were euthanized with 100 mg/kg of pentobarbital sodium, and then the tumor tissues were completely removed, weighed, and archived for photography. All animal experimental procedures were approved by the First Affiliated Hospital of University of Science and Technology of China (Approval No. 2026-re-276).

### 2.13 LPO detection

Lipid peroxidation levels in tissues were determined using a commercial LPO assay kit (BC5245, Solarbio). Briefly, approximately 0.1 g of tissue was homogenized in 1 mL of extraction solution on ice and then centrifuged at 8000 g for 10 minutes at 4 °C. The resulting supernatant was collected for subsequent analysis. Took the supernatant and placed it on ice for testing. Preheated the microplate reader for more than 30 minutes, and adjusted the wavelength to 532 nm and 600 nm. Preparation of standard solution: The standard solution was 1000 nmol/mL MDA standard solution. Diluted the standard solution with dilution solution to 20, 10, 5, 2.5, 1.25, 0.625, 0.3125, and 0.15625 nmol/mL for standby. Placed the mixture in a 100 °C water bath for 60 minutes, then cooled it in an ice bath, and centrifuged at 8000 g at room temperature for 10 minutes. Took 200 μL of the supernatant and placed it in a micro glass cuvette or 96-well plate, and measured the absorbance at 532 nm and 600 nm for each sample. Calculated ΔA= (A532 measured - A532 blank) - (A600 measured-A600 blank), and ΔA standard= (A532 standard-A532 blank) - (A600 standard-A600 blank) (standard curve, only need to perform 1–2 times for the blank tube and the control tube). Based on the concentration (x, nmol/mL) and absorbance ΔA standard (y, ΔA standard) of the standard tube, established a standard curve. According to the standard curve, substituted ΔA (y, ΔA) into the formula to calculate the sample concentration (x, nmol/mL). Calculated the LPO content based on the sample protein concentration: LPO content (nmol/mg prot) = x × V sample ÷ (Cpr × V sample) = x ÷ Cpr.

### 2.14 Statistical analysis

The data and statistical assessments conformed to the principles of experimental design and analysis. Animals were randomly assigned to experimental groups using a computer-generated randomization schedule to ensure unbiased allocation. All experiments were performed in a randomized and blinded manner: tumor volume measurements during treatment, tumor weight assessments at endpoint, and subsequent histopathological evaluations were conducted by investigators blinded to group assignments to minimize subjective bias. The results were presented as mean ± standard deviation. In order to reduce unwanted variability, all data used in this study were standardized. Statistical analyses were carried out using SPSS software. For groups with equal variances, one-way ANOVA with subsequent *post hoc* tests was utilized. A p value below 0.05 was considered statistically significant.

## 3 Results

### 3.1 ICT effectively inhibited viability and proliferation of breast cancer cells *in vitro* by ferroptosis

ICT is considered to be the main active ingredient of the herb *Epimedium* (**Figure 1A**). Studies have shown that, ICT and curcumol synergistically induce autophagy and ferroptosis in PCa cells, affecting lipid metabolism (24). In order to clarify how ICT inhibits the growth of breast cancer, this study explored the potential roles of ferroptosis and autophagy in these cells. Prior to investigating the underlying mechanisms, we first determined the optimal working concentration of ICT through dose response experiments. Cells were treated with a series of ICT concentrations (0, 5, 10, 20, 40 μmol/L) for 48 hours, and cell viability was measured by CCK-8 assay. The results showed that ICT inhibited the proliferation of both MCF-7 and MDA-MB-231 cells in a dose dependent manner, with IC50 values of 19.89 μmol/L for MCF-7 cells and 20.15 μmol/L for MDA-MB-231 cells (**Supplementary Figure 1**). Based on these results, 20 μmol/L ICT, which is close to the IC50 value and can induce significant biological effects without causing excessive cell death, was selected as the working concentration for all subsequent *in vitro* experiments. Compared to the untreated control group, cell viability was significantly reduced in both the ICT-treated group and the ICT + 3-MA group (p < 0.01). Notably, the suppressive effect of ICT was completely abrogated by co-treatment with the ferroptosis inhibitor Fer-1, indicating that the cytotoxicity of ICT is primarily mediated through the induction of ferroptosis (**Figure 1B**). Apoptosis was analyzed by flow cytometry. The apoptosis rate was significantly elevated in the ICT, ICT + 3-MA, and ICT + Fer-1 groups compared to the control (p < 0.01). Importantly, the combination of ICT with the autophagy inhibitor 3-MA resulted in a significantly higher apoptosis rate than ICT treatment alone. In contrast, concurrent administration of Fer-1 significantly attenuated the ICT-induced apoptosis (**Figure 1C**). To elucidate the mechanism of ICT-induced ferroptosis, key biochemical markers were measured. Detection of intracellular reactive oxygen species (ROS) using the DCFH-DA probe revealed markedly increased ROS levels in all ICT-treated groups compared to the control (p < 0.01), confirming that ICT induces substantial oxidative stress (**Figure 1D**). Consistent with this, a colorimetric assay showed that ICT treatment significantly increased intracellular labile iron levels (p < 0.01). This iron accumulation was further exacerbated in the ICT + 3-MA group and was partially but significantly reversed by Fer-1 co-treatment (**Figure 1E**). Western blot analysis was performed to examine the expression of related proteins (**Figures 1F, G**). In both MCF-7 and MDA-MB-231 cells, the protein level of p62 showed no statistically significant difference among all groups, suggesting that autophagic flux remained intact upon ICT treatment. However, the expression of the key ferroptosis defense proteins SLC7A11 and GPX4 was significantly downregulated by ICT. Concurrently, the LC3B-II/I ratio was markedly increased (p < 0.05), indicating activated autophagy. Strikingly, the addition of 3-MA to ICT further potentiated the downregulation of SLC7A11/GPX4 and the increase in the LC3B-II/I ratio (p < 0.05 vs. ICT group). Conversely, Fer-1 co-treatment partially but significantly rescued the expression of SLC7A11 and GPX4 from ICT-induced suppression. These protein-level data demonstrate that ICT triggers ferroptosis by inhibiting the SLC7A11/GPX4 axis and that inhibition of autophagy (by 3-MA) and ferroptosis (by Fer-1) exert opposing effects on this pathway.

### 3.2 MT1X was the key gene for ICT targeting the ferroptosis pathway to treat breast cancer

**Figure 2:**
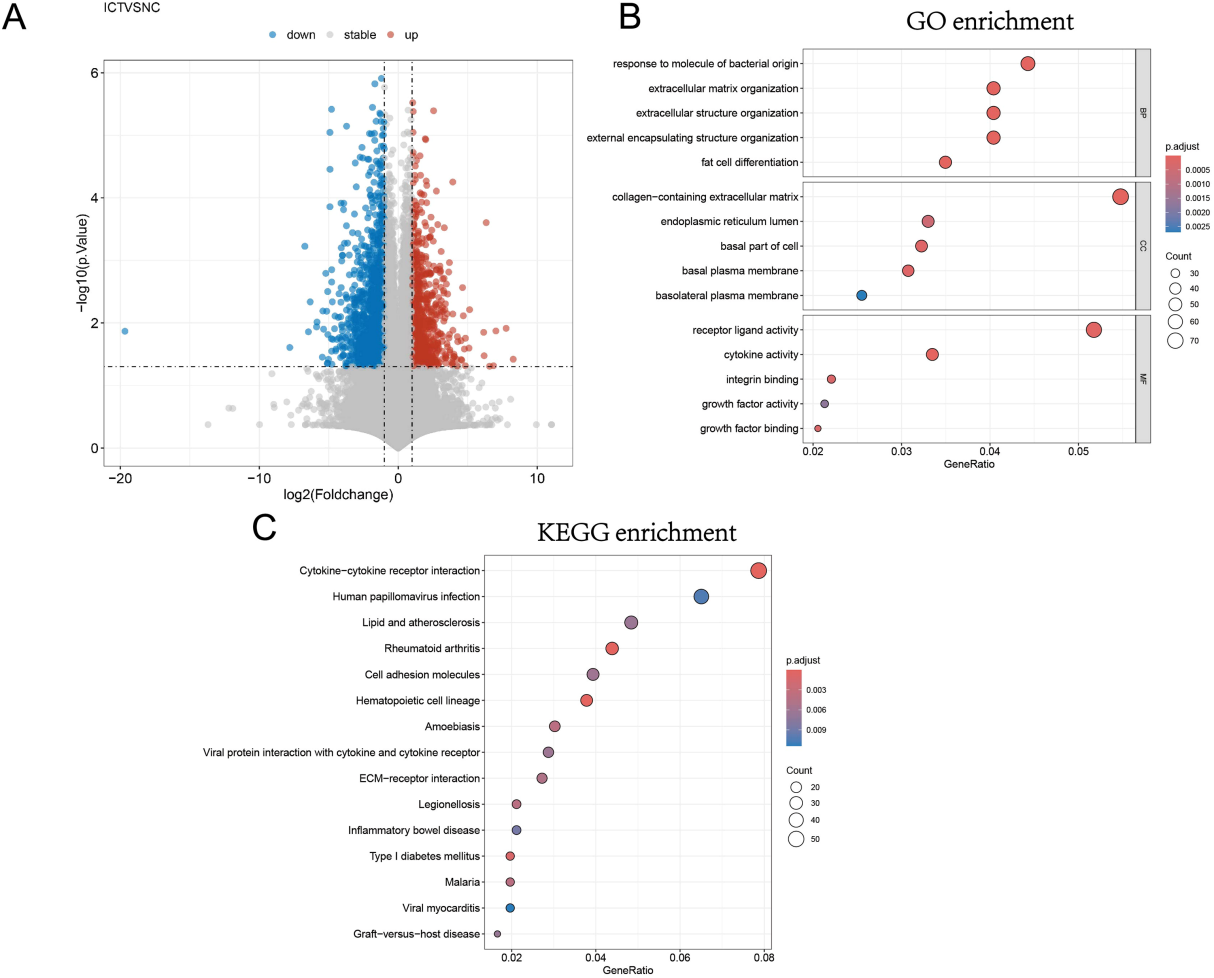
Transcriptome analysis of the effect of ICT in MD-MBA-231 cells. **(A)** Volcano plot showing genes upregulated and down reglated by RNA-seq in ICT treated MD-MBA-231 cells. **(B)** GO enrichment analysis. **(C)** KEGG enrichment analysis.

Comparison of the transcriptome data between ICT-treated breast cancer cells and untreated controls identified 2,208 differentially expressed genes (DEGs), including 813 significantly upregulated and 1,395 significantly downregulated genes. (|log_2_FC| > 1, adjusted P < 0.05; **Figure 2A**). Enrichment analysis of the DEGs for GO and KEGG revealed that the upregulated DEGs were significantly enriched in pathways related to cytokine-cytokine receptor interaction, while the downregulated DEGs were primarily enriched in cancer-related pathways (**Figures 2B, C**).

**Figure 3:**
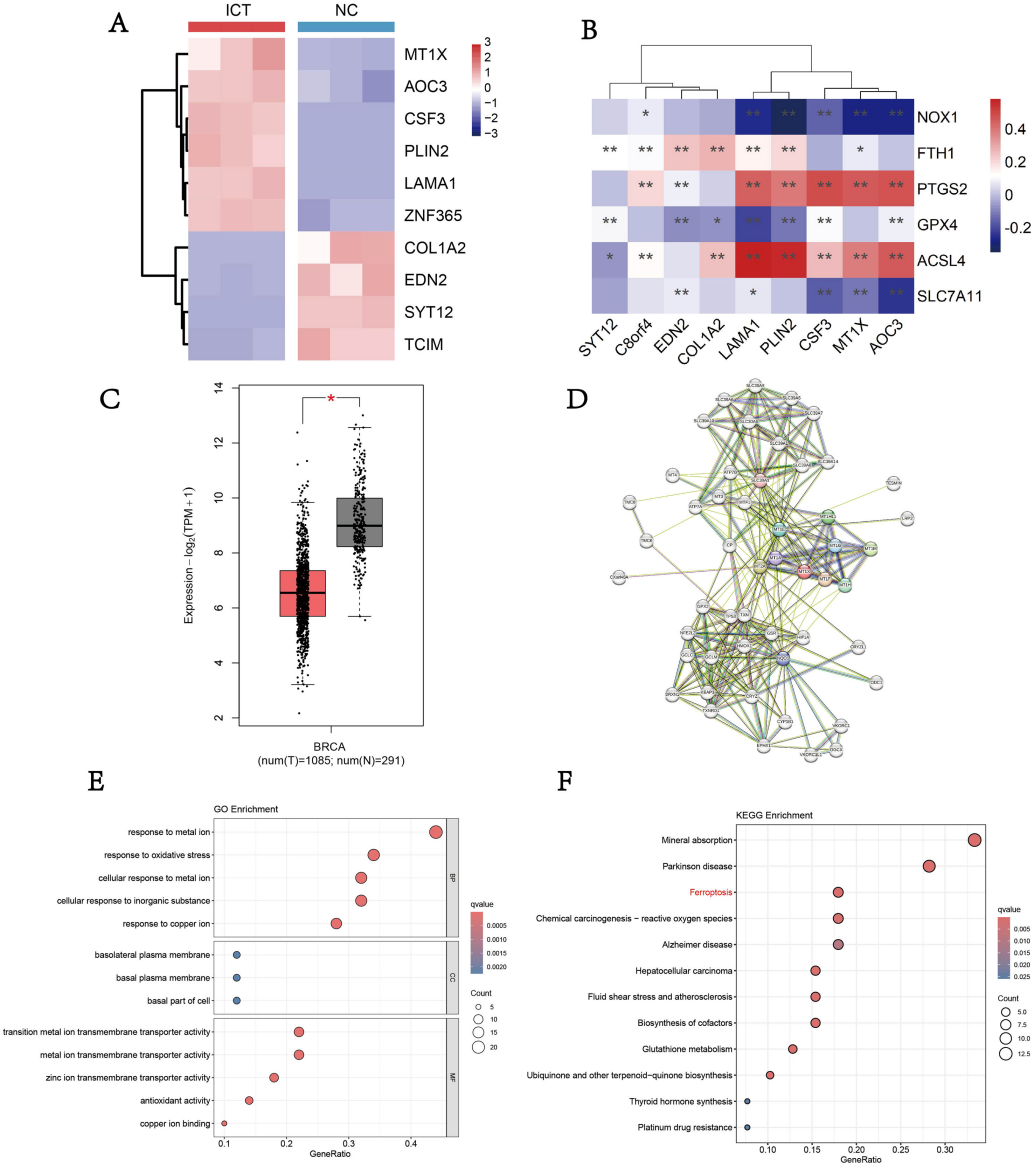
Bioinformatics analysis revealed that MT1X might be a downstream target of ICT. **(A)** Heatmap are used to represent the expression of genes in transcriptome sequencing, with red indicatig up regulation and blue indicating down-regulation. **(B)** Pearson correlation analysis of DEGs and genes related to ferroptosis, r>0.3, P<0.05. **(C)** Differential expression analysis of MT1X in clinical data of breast cancer. **(D)** PPI analysis of the interacting protein of MTIX. **(E, F)** GO and KEGG enrichment analysis.

Screening of the DEGs based on fold change identified five genes (MT1X, AOC3, LAMA1, PLIN2, and CSF3) that were significantly upregulated following ICT treatment, and four genes (TCIM, EDN2, COL1A2, and SYT12) that were significantly downregulated (**Figure 3A**). Given that previous research demonstrated that ICT could induce ferroptosis in breast cancer cells, we performed correlation analysis between the screened DEGs and known ferroptosis-related genes. This analysis showed that these DEGs exhibited significant correlations with ferroptosis-related genes (**Figure 3B**; r > 0.3, P < 0.05). Clinical data analysis of the nine DEGs was conducted using the online databases GEPIA2 and TCGA. The results indicated that MT1X was significantly downregulated in breast cancer tissues compared to normal tissues (**Figure 3C**). Metallothioneins (MTs) are a family of cysteine-rich, low-molecular-weight intracellular proteins capable of binding metal ions. They play roles in regulating metal ion homeostasis, responding to oxidative stress, and participating in the progression of various cancers (25, 26). Literature review indicated that MT1X, an isoform of the MT protein family, impeded cell cycle progression, promoted apoptosis of liver cancer cells, and inhibited tumor growth and lung metastasis *in vitro* and *in vivo* upon overexpression (27). Clinical data analysis of oral cancer demonstrated that MT1X was significantly downregulated in oral squamous cell carcinoma (OSCC), and its elevated expression was negatively correlated with OSCC metastasis (28). However, the role of MT1X in breast cancer remained unexplored. Therefore, we focused on the MT1X gene. Using the STRING database (version 12.0), we retrieved the top 50 proteins interacting with the MT1X protein and constructed a protein-protein interaction (PPI) network. Subsequent KEGG and GO enrichment analysis of these 51 proteins revealed that MT1X was closely associated with the response to metal ions and ferroptosis-related pathways (**Figures 3D–F**).

### 3.3 ICT promoted ferroptosis in breast cancer cells through MT1X

**Figure 4:**
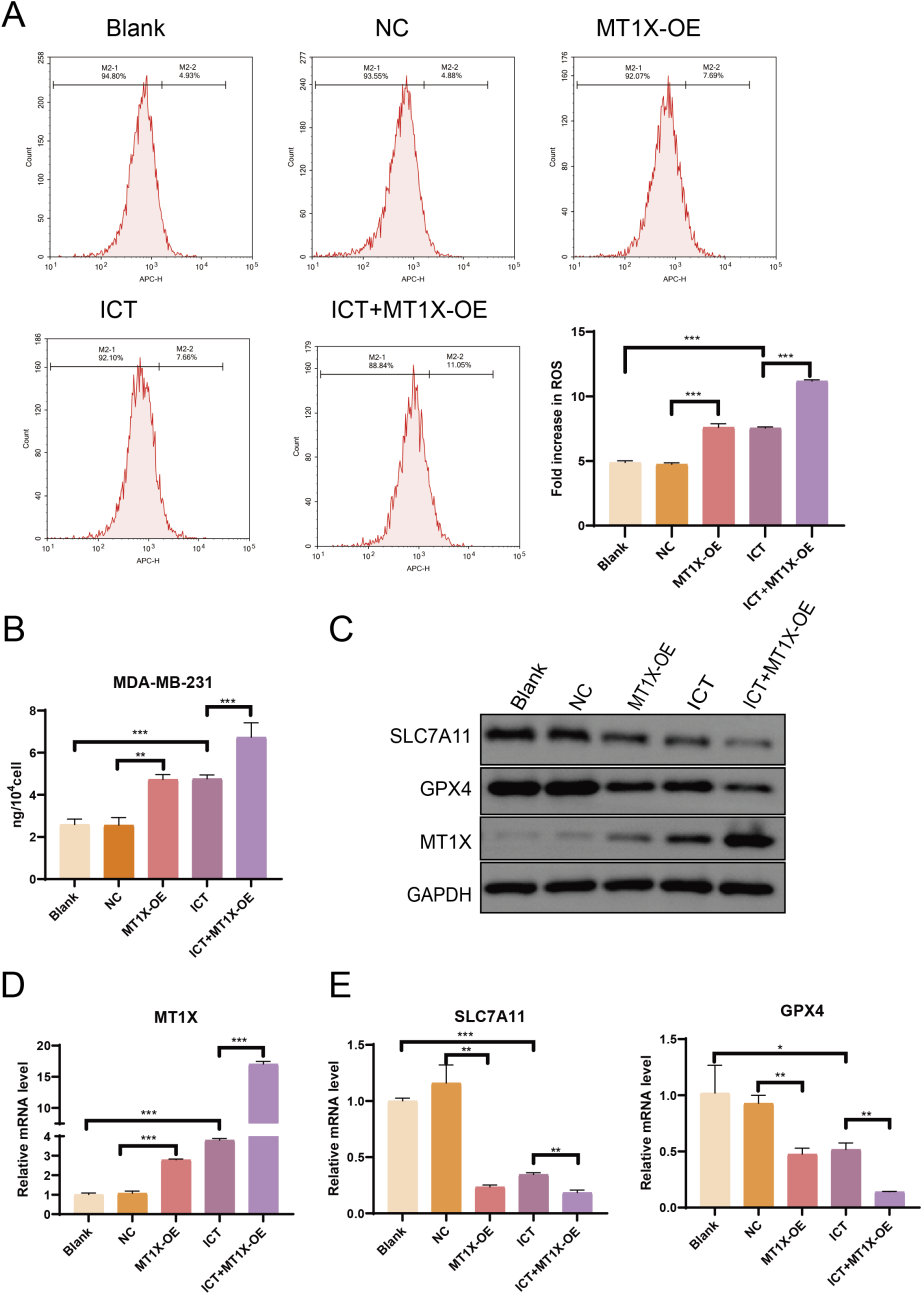
ICT upregulates MT1X expression in breast cancer cells to influence iron death process. **(A)** After staining cells with DCFH-DA fluorescent probe, we measured reactive oxygen species (ROS) levels across experimental subgroups using flow cytometry. **(B)** Folin-phenolic iron ion colorimetry (phenanthroline colorimetry) quantified Fe²^+^ content. **(C–E)** Quantitative PCR (qPCR) and Western blotting were performed to assess the expression levels of MT1X and key ferroptosis-related genes (GPX4, SLC7A11). *P < 0.05; **P < 0.01; ***P < 0.001.

Ferroptosis was defined as an iron-dependent regulated form of cell death induced by the accumulation of ROS within the cellular matrix, with elevated lipid peroxidation and iron accumulation serving as its distinctive biochemical features (29). To elucidate the relationship between MT1X and ferroptosis during ICT treatment of breast cancer, we had first constructed a lentiviral vector for MT1X overexpression. Oxidative stress and iron metabolism detection showed that compared with the NC group, the intracellular Fe²^+^ concentration and ROS levels were significantly increased in the MT1X-OE group. Moreover, Fe²^+^ and ROS levels were further elevated in the ICT + MT1X-OE group compared with the ICT group (P<0.001; **Figures 4A, B**). To elucidate the regulatory mechanism of ICT and MT1X on ferroptosis, the expression of ferroptosis-related genes was examined using qPCR and WB experiments. The results indicated that ICT significantly upregulated the expression of MT1X. Relative to the NC group, the MT1X-OE group exhibited significantly lower SLC7A11 and GPX4 expression levels, which decreased further in the ICT + MT1X-OE group compared to either the ICT or MT1X-OE group alone (P<0.01; **Figures 4C–E**). These results demonstrate that ICT upregulates MT1X while inhibiting SLC7A11 and GPX4 activity, leading to increased iron accumulation, oxidative stress, and subsequent ferroptosis activation in breast cancer cells.

### 3.4 ICT suppressed breast cancer cell survival by modulating ferroptosis via MT1X

**Figure 5:**
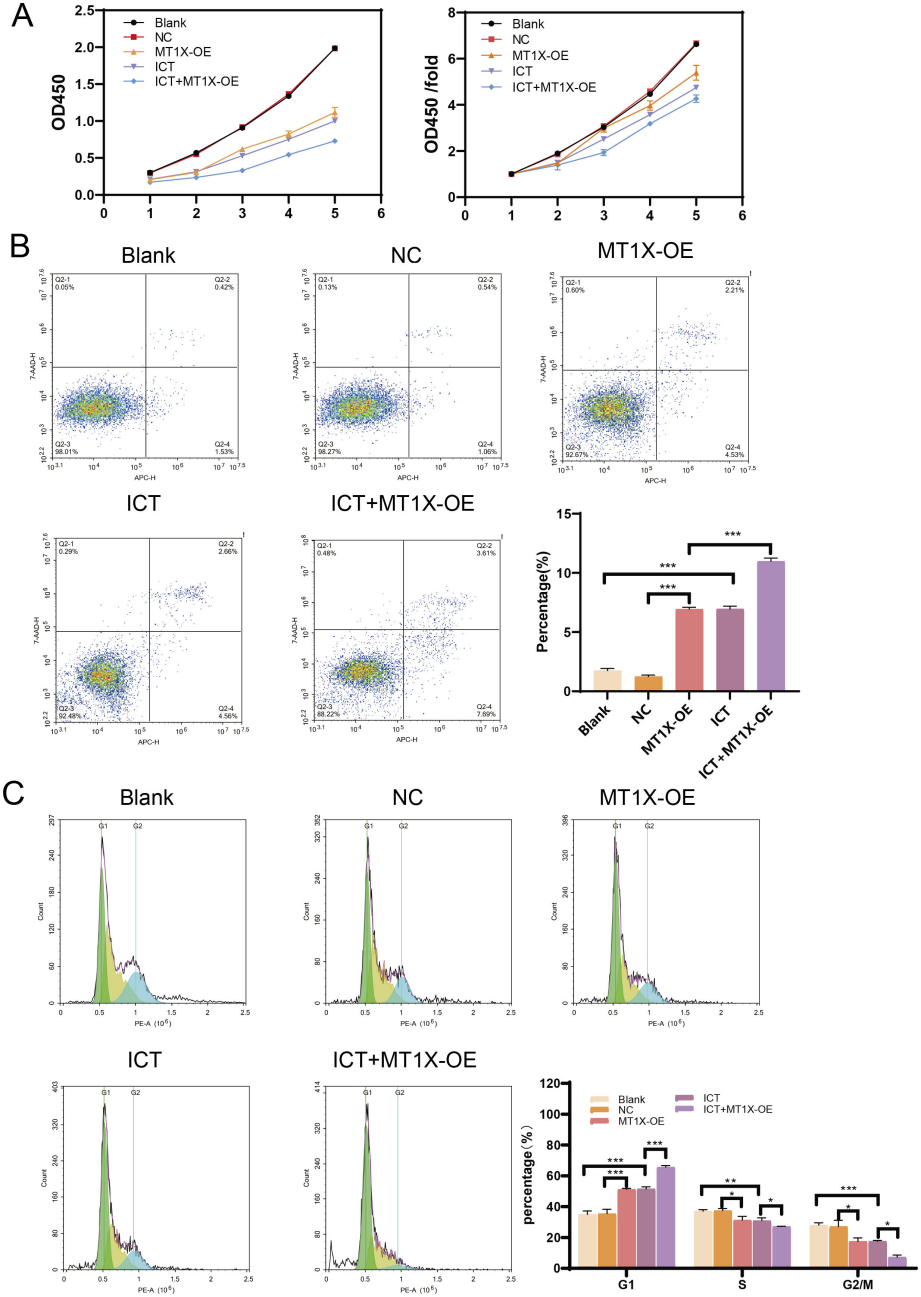
ICT regulated ferroptosis via MT1X, inhibiting viability, apoptosis, and cell cycle progression in breast cancer cells. **(A)** CCK-8 assay for cell proliferation ability. **(B)** Flow cytometry quantified apoptotic cells across all groups. **(C)** The flow cytometric analysis revealed distinct cell cycle distributions across experimental groups. *P < 0.05; **P < 0.01; ***P < 0.001.

Based on the aforementioned evidence, we have demonstrated that ICT modulates the ferroptosis process in breast cancer cells through MT1X. Genetic or pharmacological activation of ferroptosis has been shown to significantly suppress neoplastic cell proliferation/growth, invasiveness, clonogenic efficiency, and anchorage-independent growth, whereas ferroptosis inhibition can conversely reverse these effects (30). The CCK-8 assay was utilized to assess cell proliferation. Compared with the NC group, the MT1X-OE group exhibited significantly diminished proliferative capacity. Furthermore, the ICT + MT1X-OE group showed a more pronounced inhibitory effect on proliferation than the ICT group alone, indicating that MT1X overexpression synergistically amplified ICT’s suppressive impact on breast cancer cell proliferation (P < 0.05; **Figure 5A**). Apoptosis assays revealed a significantly higher proportion of apoptotic cells in the MT1X-OE group than in the NC group, with the ICT + MT1X-OE group also demonstrating elevated apoptosis relative to the ICT group (P < 0.001; **Figure 5B**). Cell cycle analysis indicated that MT1X overexpression increased the G1 phase population while reducing the S and G2/M phase proportions compared to the Blank group. The ICT + MT1X-OE group displayed more pronounced G1 phase arrest than the ICT group, suggesting that ICT treatment or MT1X overexpression regulated cell cycle progression to suppress tumor growth (**Figure 5C**).

### 3.5 ICT inhibited *in vivo* tumor growth and ferroptosis in breast cancer through MT1X

**Figure 6:**
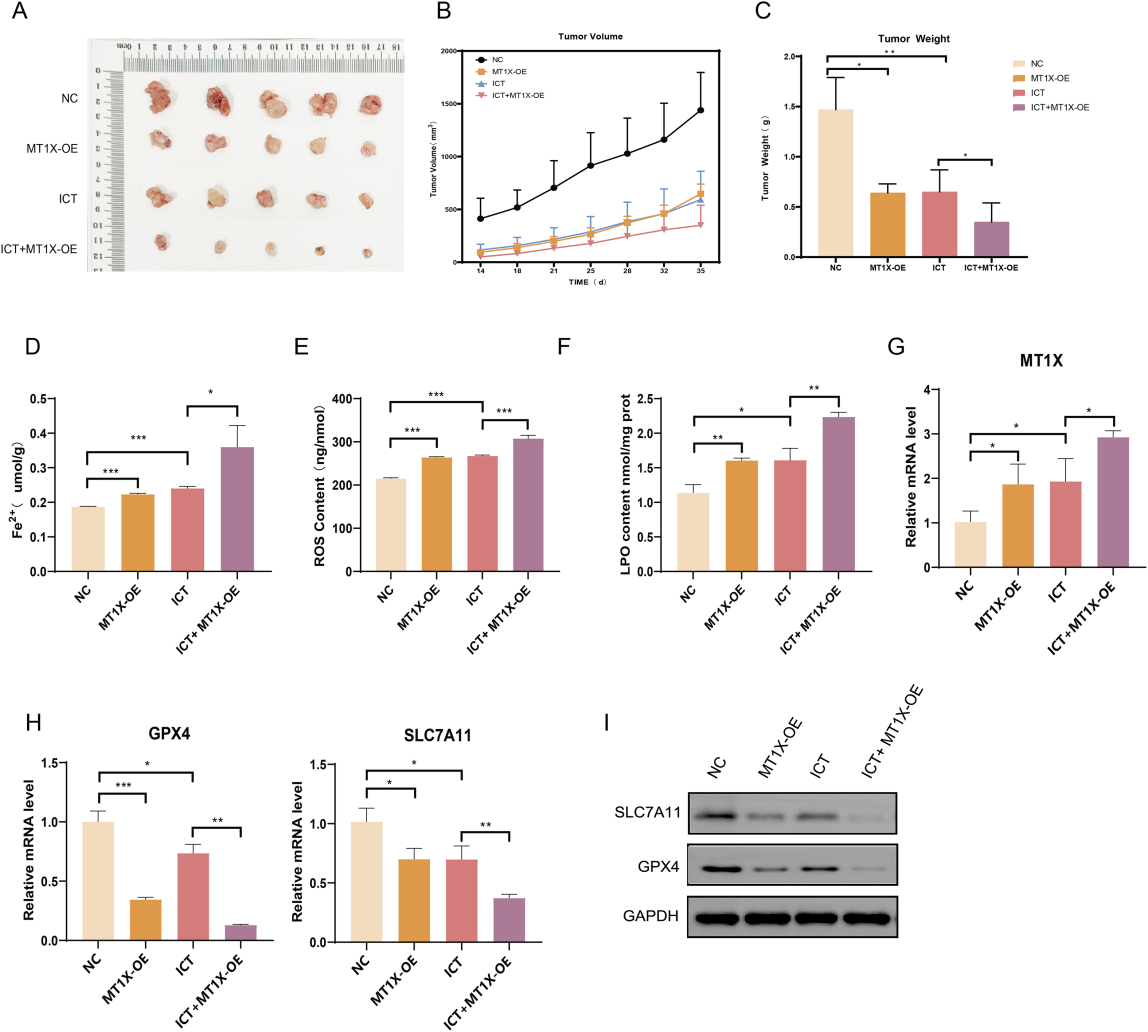
ICT upregulates MT1X expression to modulate ferroptosis in a breast cancer *in vivo* model. **(A)** Visual image of xenograft of nude mice. **(B)** Tumor weight xenograft of nude mice. **(C)** Tumor volume size of xenograft of nude mice. (Calculation formula V = W^2^ × L/2). **(D)** Folin-phenolic iron ion colorimetry (phenanthroline colorimetry) quantified Fe²^+^ content. **(E)** The intracellular ROS levels in tumor tissues were measured using the DCFH-DA fluorescent probe. **(F)** The lipid peroxide (LPO) detection kit measured lipid peroxidation levels in tumor tissues quantitatively. **(G, H)** Quantitative PCR (qPCR) was performed to assess the mRNA expression levels of MT1X and key ferroptosis-related genes (GPX4 and SLC7A11). **(I)** Western blotting was performed to assess the expression levels of key ferroptosis-related proteins (GPX4, SLC7A11). *P < 0.05; **P < 0.01; ***P < 0.001.

To investigate the effects of ICT and MT1X on breast cancer progression *in vivo*, tumor formation assays were conducted in nude mouse models. Compared with the NC group, both the MT1X-OE group and the ICT-treated group exhibited significantly reduced tumor weight and volume (P < 0.05). Notably, the ICT + MT1X-OE group demonstrated a more profound inhibitory effect on tumor growth than ICT monotherapy (P < 0.05; **Figures 6A–C**). Analysis of ferroptosis-related metabolic markers revealed significantly elevated levels of Fe²^+^, ROS, and lipid peroxides (LPO) in tumor tissues from the MT1X-OE and ICT groups compared to the NC group. Importantly, the combined application of ICT and MT1X overexpression synergistically enhanced the upregulation of these ferroptosis markers (P < 0.05 versus ICT alone; **Figures 6D–F**). At the molecular level, qPCR and Western blot analyses showed that ICT treatment markedly increased MT1X expression at both transcriptional and translational levels relative to the NC group, while MT1X-overexpression substantially suppressed SLC7A11 and GPX4 expression. The combination of ICT with MT1X overexpression produced a synergistic inhibitory effect on SLC7A11 and GPX4 expression beyond that achieved by ICT alone (**Figures 6G–I**). Collectively, these findings indicated that ICT induced ferroptosis by upregulating MT1X expression, which attenuated the SLC7A11/GPX4-mediated defense mechanism, ultimately inhibiting breast cancer progression *in vivo*.

### 3.6 ICT promoted NF-κB activation via MT1X in breast cancer

**Figure 7:**
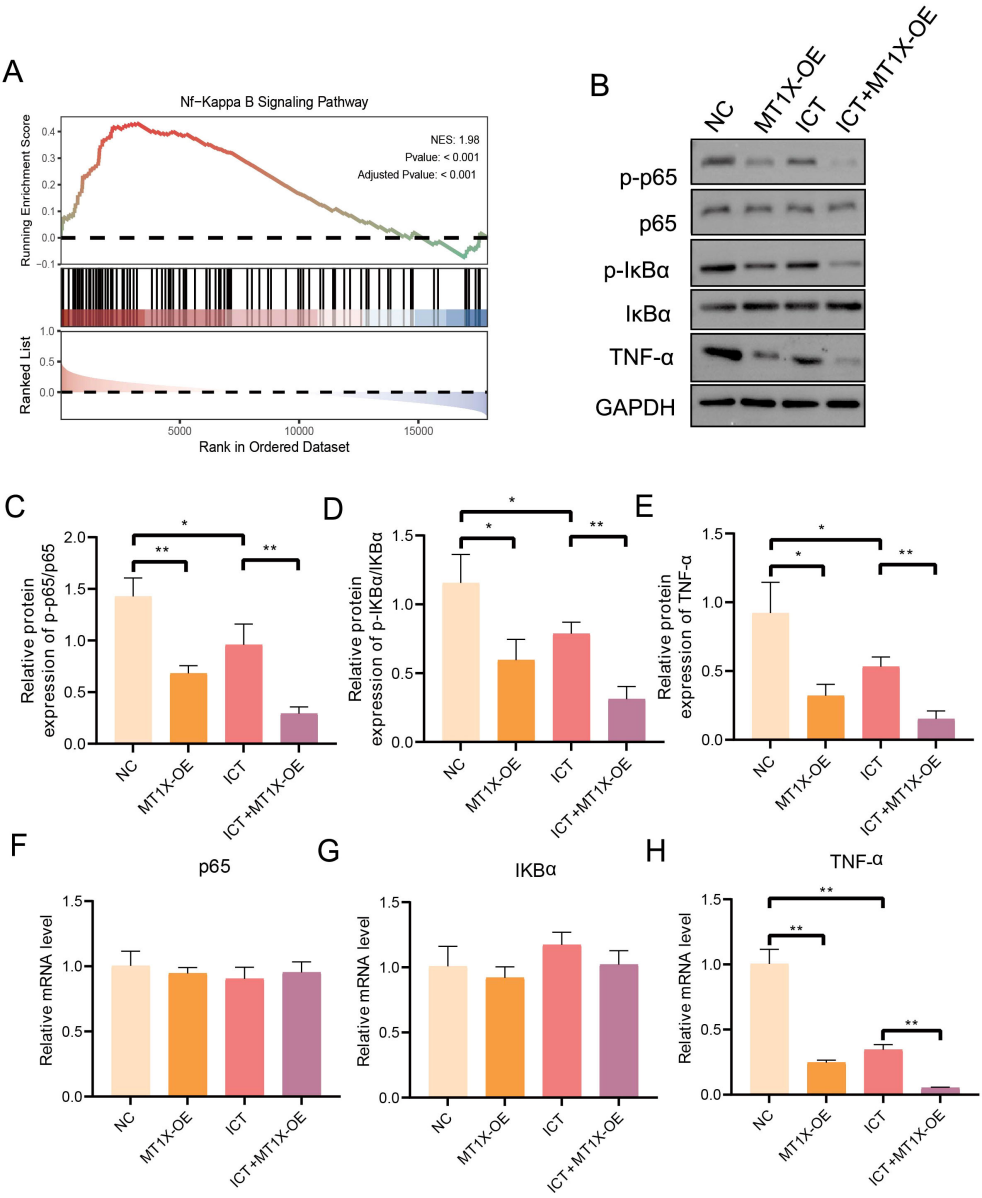
ICT regulated the NF-κB signaling pathway via MT1X. **(A)** GSEA enrichment analysis of the downstream targets of MT1X. **(B–E)** Western blot analysis of the expression of p-IKBα, p-p65 and TNF-α proteins. **(F–H)** qPCR analysis of the expression of p65, IKBα and TNF-α. *P < 0.05; **P < 0.01.

To dissect downstream mechanisms regulated by MT1X, we performed transcriptomic data mining and Gene Set Enrichment Analysis (GSEA) focused on MT1X-associated gene sets. Results revealed significant enrichment of MT1X in the classical NF-κB signaling pathway gene set (**Figure 7A**). qPCR and Western blot analyses of key NF-κB pathway molecules in ICT/MT1X-co-treated tumor tissues showed that although total IκBα and p65 protein/mRNA levels remained unchanged, phosphorylated IκBα (p-IκBα) and p-p65 were significantly downregulated in the ICT group. The ICT+MT1X-OE combination further reduced p-IκBα and p-p65 expression (P < 0.01; **Figures 7B–D**, **F, G**). Simultaneously, both MT1X-OE and ICT groups exhibited significantly decreased TNF-α protein/mRNA expression versus the NC group. Moreover, TNF-α expression was further downregulated in the ICT+MT1X-OE group compared to ICT monotherapy (P < 0.01; **Figures 7E, H**). These findings indicated that MT1X inhibited IκBα phosphorylation (p-IκBα), reduced its binding to NF-κB p65, consequently diminishing p65 nuclear translocation and phosphorylation (p-p65), ultimately downregulating pro-cancer genes including TNF-α.

## 4 Discussion

Plant-derived natural bioactive compounds represented a critical source of potential antitumor agents (31). Icaritin (ICT), the principal active component of Epimedium, regulated key oncogenic processes including proliferation, apoptosis, invasion, and metastasis (20, 32). This study demonstrated for the first time that ICT exerted dual antitumor effects by upregulating MT1X expression: it concurrently promoted ferroptosis in breast cancer cells and suppressed NF-κB pathway activation, ultimately inhibiting cancer cell viability. These findings provided novel insights into ICT’s anticancer mechanisms and revealed MT1X’s pivotal role as a molecular bridge connecting ferroptosis and NF-κB signaling.

Our findings demonstrate that ICT significantly suppresses the proliferation of both MCF-7 and MDA-MB-231 cells. This inhibitory effect was completely abrogated by the ferroptosis inhibitor ferrostatin-1 (Fer-1), whereas co-treatment with the autophagy inhibitor 3-methyladenine (3-MA) potentiated the cytotoxicity of ICT (**Figure 2B**), indicating a central role of ferroptosis in ICT-induced cell death. This is consistent with previous studies on apigenin (33) and artemisinin derivatives (34), which also act through ferroptosis pathways. Further supporting this mechanism, colorimetric iron assays revealed that ICT combined with 3-MA promoted intracellular Fe²^+^ accumulation in breast cancer cells, an effect that was reversed by the addition of Fer-1 (**Figure 2E**). Recent studies suggest that lysosome-mediated protective autophagy can suppress ferroptosis in cancer cells by removing damaged or oxidized lipid peroxides (LPO) (35). Therefore, we speculate that upon ICT treatment, breast cancer cells may activate protective autophagy as a survival response, potentially through the clearance of ICT-damaged mitochondria (thereby reducing ROS production) or peroxidized lipids, to maintain intracellular homeostasis and counteract ICT-induced ferroptotic stress (36) (37).

From a ferroptosis perspective, ICT inhibited the SLC7A11/GPX4 axis and induced ROS and iron accumulation, consistent with canonical ferroptosis mechanisms (38). As the primary cystine/glutamate antiporter, SLC7A11 downregulation disrupted glutathione synthesis and compromised antioxidant defenses (39). Although Ferrostatin-1 (Fer-1) partially restored SLC7A11/GPX4 expression, the incomplete rescue suggested additional regulatory pathways beyond this axis. Post-transcriptional regulation of iron metabolism genes (e.g., FTH1, FTL) or ferritinophagy activation might also have contributed (40), warranting further investigation. Transcriptomic analysis identified MT1X as a critical downstream effector of ICT. As a metallothionein family member, MT1X overexpression potentiated ICT-induced suppression of SLC7A11/GPX4 and enhanced ROS and Fe²^+^ accumulation. Notably, the levels of ROS and Fe²^+^ in the MT1X-OE group were significantly higher than those in the ICT treatment group alone, suggesting that MT1X might promote the ferroptosis process by amplifying the threshold of oxidative damage (41). Clinical correlation analysis demonstrated that MT1X was significantly downregulated in breast cancer tissues, which is consistent with its expression characteristics in gastric cancer (42), and liver cancers (43), suggesting a broader tumor-suppressive function. Its expression correlated positively with ferroptosis markers (r > 0.3), implicating epigenetic regulation, such as promoter methylation.

Gene Set Enrichment Analysis (GSEA) identified a significant functional association between MT1X and the NF-κB and PI3K-Akt signaling pathways. Western blot analysis demonstrated that overexpression of MT1X markedly suppressed the phosphorylation of IκBα and p65, indicating impaired nuclear translocation of NF-κB and a consequent downregulation of pro-survival signaling. *In vitro* experiments revealed that MT1X overexpression inhibited cell viability while promoting G1 phase cell cycle arrest. This finding highlighted the critical role of the MT1X/NF-κB axis in ICT’s anti-breast cancer activity. This study provided the first evidence that MT1X exerted tumor-suppressive effects in breast cancer through NF-κB pathway regulation, thus expanding our understanding of MT1X function. The NF-κB signaling pathway played a pivotal role in tumorigenesis and progression (44). By suppressing the NF-κB pathway, MT1X not only directly inhibited breast cancer cell proliferation but also synergized with ICT-induced ferroptosis, further enhancing the anti-tumor efficacy.

*In vitro* and *in vivo* models confirmed ICT’s tumor growth inhibitory effects, which were further augmented by MT1X overexpression. Compared to conventional chemotherapeutics, ICT demonstrated distinct therapeutic advantages: (1) multi-target modulation that mitigated drug resistance (32); (2) induction of tumor cell ferroptosis while preserving normal cell viability (45); and (3) modulation of key protein phosphorylation in the NF-κB pathway, resulting in pathway activation inhibition (27).However, the exact mechanism of MT1X mediated NF-κB suppression remains unclear. As a classic metallothionein, MT1X exerts antioxidant and metal homeostatic functions. It may inhibit NF-κB signaling through two conserved pathways: scavenging ROS to block NF-κB nuclear translocation, and releasing zinc ions to modulate IKK complex activity (46). We validated the role of MT1X in ICT-mediated anti-breast cancer effects through gain-of-function experiments. However, loss-of-function validation via siRNA/shRNA knockdown or CRISPR/Cas9 knockout remains to be performed, and compensatory pathways cannot be entirely excluded. Whether MT1X serves as an essential mediator of ICT-induced ferroptosis will be a priority in our follow-up studies.

We demonstrate that ICT-induced MT1X upregulation in breast cancer development simultaneously potentiates ferroptotic cell death and reduces phosphorylation in the NF-κB signaling cascade. However, the critical bridging molecules connecting MT1X-mediated NF-κB suppression and ferroptosis activation remain uncharacterized. Furthermore, while validated in cellular and animal models, the mechanism requires clinical sample verification to establish its *in vivo* relevance and therapeutic potential.

## 5 Conclusion

This study reveals that ICT inhibits breast cancer progression by upregulating MT1X expression, triggering ferroptosis, and regulating the NF-κB pathway, offering potential therapeutic targets for breast cancer intervention. The integration of traditional Chinese medicine monomers with epigenetic regulation represents a key frontier in tumor treatment, highlighting the translational potential of natural compounds in precision oncology.

## Data Availability

The datasets presented in this study can be found in online repositories. The names of the repository/repositories and accession number(s) can be found below: https://www.ncbi.nlm.nih.gov/, PRJNA1356289.
