# Supplementary material for: Icaritin inhibits breast cancer through activating MT1X to promote ferroptosis and synergizing with NF-κB pathway suppression
Source: Front Oncol. 2026 Jul 10;16:1868122. doi: 10.3389/fonc.2026.1868122 (PMC13395711; doi:10.3389/fonc.2026.1868122)
Supplement: Supplementary file 1 [file DataSheet1.docx]

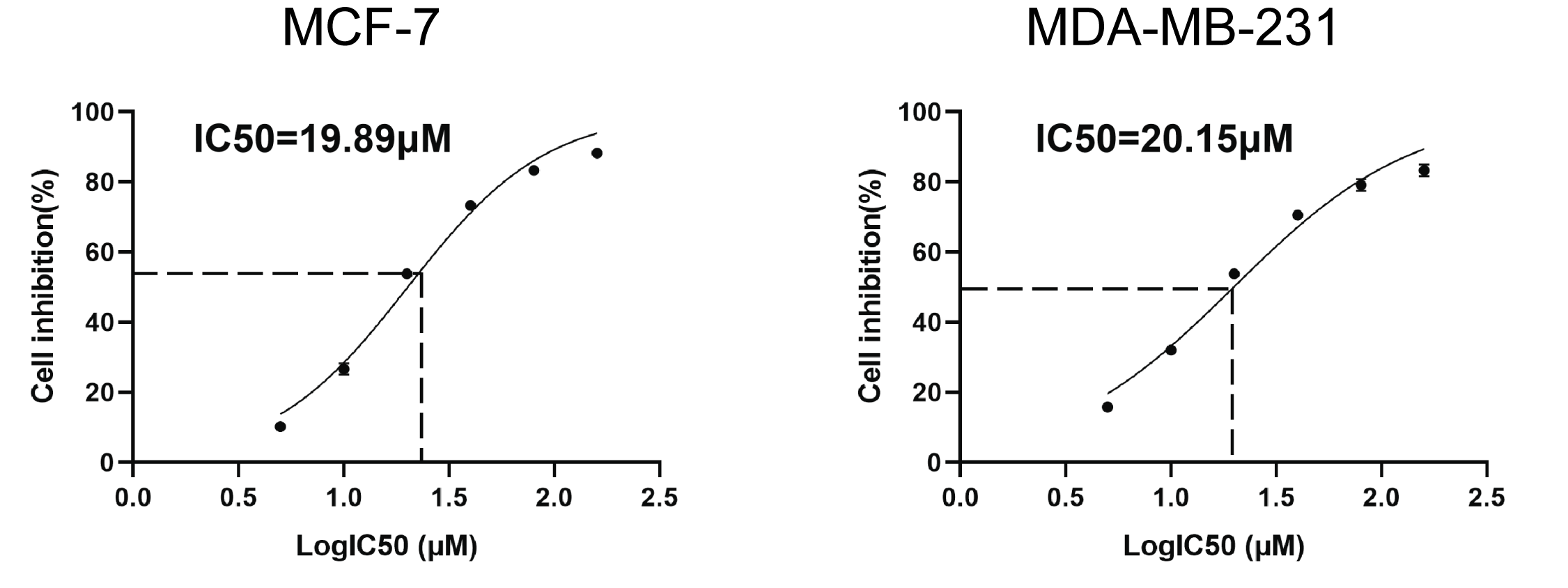


Supplementary Figure S1. Dose-response curves of icaritin (ICT) in breast cancer cells. MCF-7 and MDA-MB-231 cells were treated with a series of ICT concentrations for 48 hours. Cell viability was measured by CCK-8 assay, and cell inhibition rates were calculated based on the viability data. The sigmoidal dose-response curves were fitted using GraphPad Prism 8.0 software. Left panel: MCF-7 cells; Right panel: MDA-MB-231 cells. The calculated IC50 values were 19.89 μM for MCF-7 cells and 20.15 μM for MDA-MB-231 cells. All experiments were performed in triplicate and repeated three times independently.
